# Effect of Dietary Supplementation with Coarse or Extruded Oat Hulls on Growth Performance, Blood Biochemical Parameters, Ceca Microbiota and Short Chain Fatty Acids in Broiler Chickens

**DOI:** 10.3390/ani10081429

**Published:** 2020-08-15

**Authors:** Deborah Adewole

**Affiliations:** Department of Animal Science and Aquaculture, Faculty of Agriculture, Dalhousie University, Truro, NS B2N 5E3, Canada; deborah.adewole@dal.ca; Tel.: +1-902-893-8023

**Keywords:** broiler chickens, coarse oat hulls, extrusion, gut microbiota, short-chain fatty acids

## Abstract

**Simple Summary:**

Oat hull (OH) is an excellent source of insoluble fiber, which may improve chickens’ gastrointestinal health by promoting the growth of beneficial bacteria. Extrusion may modify OH fiber fractions resulting in a differential effect on gut microbiota. Thus, the purpose of this study was to determine the effect of dietary supplementation with coarse or extruded OH on growth performance, blood biochemistry and cecal short-chain fatty acids (SCFA) and microbiota of broiler chickens. Broiler chickens were assigned four diets: a corn-wheat-soybean-based diet, an antibiotics diet, 3% coarse OH (COH), and 3% extruded OH (EOH) supplemented diets. Birds fed COH and EOH had reduced BWG, but there was no diet effect on FCR. There was a significant increase in relative gizzard weight among birds that were fed COH. Dietary treatments had no effect on blood biochemical parameters and SCFA concentration. Cecal microbial composition of chickens was mostly comprised of Firmicutes and Tenericutes. Seven bacteria species that were differentially abundant among treatments were identified

**Abstract:**

The purpose of this study was to determine the effect of dietary supplementation with coarse or extruded oat hulls on growth performance, blood biochemistry, cecal microbiota, and short chain fatty acids (SCFA) in broiler chickens. Chickens were randomly allotted to four dietary treatments consisting of a corn-wheat-soybean meal-based diet (Basal), Basal + Bacitracin methylenedisalicylate (BMD), Basal +3% coarse OH (COH), and basal +3% extruded OH (EOH). Feed intake (FI), body weight gain (BWG), and feed conversion ratio (FCR) were recorded weekly. On day 36, eight chickens/treatment were euthanized, blood samples were collected, and organ weights were determined. Cecal digesta samples were collected for the determination of SCFA concentration and microbial DNA sequence. Data were subjected to ANOVA using the mixed procedure of SAS. Alpha diversity was estimated with the Shannon index, and the significance of diversity differences was tested with ANOVA. Birds fed COH and EOH had reduced (*p* < 0.05) BWG, but there was no effect of treatment on FCR. There was a significant increase (*p* = 0.0050) in relative gizzard empty weight among birds that were fed COH, compared to the other treatments. Dietary treatments had no effect on blood biochemical parameters and SCFA concentration. Cecal microbial composition of chickens was mostly comprised of Firmicutes and Tenericutes. Seven OTUs that were differentially abundant among treatments were identified. In conclusion, supplementation of broiler chickens’ diets with 3% COH or EOH did not affect the FCR, blood biochemical parameters and SCFA concentration, but modified few cecal microbiota at the species level. Dietary supplementation with COH but not EOH significantly increased the relative gizzard weight.

## 1. Introduction

Oat hulls (OH) have high content of insoluble fiber [1,2] and their moderate inclusions in broiler chickens’ diets have resulted in improvement in nutrient digestibility, and gizzard and intestinal functionality [3,4,5]. Ndou et al. [6] reported that dietary supplementation with OH modulated gastrointestinal microbiota in pigs. Supplementing broiler chickens’ diets with OH, therefore, has the potential to enhance gut microbiota and short-chain fatty acids production, thus increasing gut barrier integrity. The structure and mode of feeding of OH fiber have been reported to play an essential role in enhancing intestinal function and modifying the composition and quantity of the microbial population in the chicken gastrointestinal tract [7]. Our previous study [8] showed that inclusion of 3% fine particle-sized OH has the potential to enhance growth performance and carcass weight of broiler chickens while OH fed as free-choice did not. Gracia et al. [9] reported reduced cecal Campylobacter jejuni colonization when 5% OH was fed to broiler chickens in mash form compared to those fed in pelleted form. Hetland and Svihus [10] reported a faster feed passage with the inclusion of coarsely ground OH, but no effect of finely ground OH was found. Sacranie et al. [11] reported that intermittent feeding of OH showed improvement in nutrient digestibility compared to regular feeding. Coarse OH has high lignin content, which may limit microbial fermentation in the hindgut [12]. Extrusion, a thermal and mechanical processing technique, which combines high pressure with high temperatures, has been known to improve the nutritive value of feed ingredients [13,14] and is widely used to improve the functional properties of food. Extrusion cooking of pea hulls caused a redistribution from insoluble dietary fiber to soluble dietary fiber, indicating a partial solubilization of fiber, without extensive degradation of the polymeric structure [15]. Sobota and Rzedzick [16] also reported that extrusion resulted in decreased content of neutral detergent fiber (NDF), acid detergent fiber (ADF) and cellulose fractions of pea hulls and caused a reduction in the content of insoluble dietary fiber and an increase in the content of soluble dietary fiber fractions. Extrusion cooking significantly increased soluble dietary fiber content of lupin seed coat, from 29 g/kg dry basis to 90 g/kg dry basis, while decreasing insoluble dietary fiber content from 899 g/kg to 829 g/kg [17]. Extrusion cooking influences the nature of feed components by changing the physical (particle size), chemical (starch gelatinization, inactivation of antinutrients) and nutritional (nutrient digestibility) properties [18]. Although OH does not have significant amounts of starch and protein [2,8], its extrusion may change its particle size and cause a redistribution of its fiber components, thereby creating a differential effect on gut microbiota than coarse OH. Various dietary fiber types have been reported to differentially affect blood biochemical parameters [19,20,21,22] with soluble fiber observed to reduce the concentrations of triglycerides, phospholipids, and low density lipoprotein (LDL) cholesterol in the blood [23,24,25]. The objective of this study, therefore, was to determine the effect of coarse and extruded OH on growth performance, blood biochemical characteristics, and cecal SCFA and microbiota in broiler chickens.

## 2. Materials and Methods

The Dalhousie University Animal Use and Care Committee approved the experimental procedures for this study (ethical code 2020-011), and chickens were cared for following the guidelines of the Canadian Council on Animal Care [26].

### 2.1. Birds and Housing

Mixed-sex broiler chickens (Ross 308) were obtained from a commercial source and were weighed in groups of 26 birds and assigned to floor pens (0.93 m × 2.14 m), at a stocking density of 0.076 m^2^/bird. Room temperature was monitored daily and was gradually reduced from 30 to 22.6 °C from d 0 to 36. The lighting program was set to produce 18 h of light and 6 h of darkness throughout the experimental period, and illumination was gradually reduced from 20 Lux on d 0 to 5 Lux on d 36.

### 2.2. Oat Hulls, Diets and Experimental Design

The OH used in this study was donated by Grain Millers, Yorton, Saskatchewan, Canada. The extruded OH was prepared by passing the OH through a Kahl OEE8 extruder (Amandus Kahl GmbH and Co. KG, Reinbek, SH, Germany) at a barrel temperature set at 100 °C. After extrusion, the OH was then air-dried for 4 h. Four dietary treatments were assigned to 24 pens in a completely randomized design. The dietary treatments consisted of a corn-soybean meal-wheat based diet (Basal), Basal + Bacitracin methylenedisalicylate (BMD), Basal +3% coarse OH (COH), and basal +3% extruded OH (EOH). The COH and EOH treatments had reduced the calculated energy level as a result of diet dilution with OH. Birds were fed using a phase-feeding program that consisted of starter phase (d 0 to 14), grower phase (d 14 to 24), and finisher phase (d 24 to 39), and diets were fed in pelleted and crumbled form during the starter phase and in pelleted form during the grower and finisher phases. The analyzed compositions of the OH samples are shown in Table 1. The ingredient and nutritional compositions of the diets in phases 1 to 3 are presented in Table 2.

### 2.3. Measurements and Sampling

Body weight (BW) and feed intake (FI) were determined on a pen basis on d 8, 15, 22, 28, and 36, and mortality was recorded daily to calculate body weight gain (BWG) and feed conversion ratio (FCR). On day 36, one chicken was randomly selected from each pen and then they were individually weighed and euthanized by electrical stunning and exsanguination. Blood samples were collected from each bird into 5 mL heparinized tubes for blood biochemistry assay. After slaughter, empty weights of gizzard and ceca were determined. The weights of bursa of Fabricius and spleen were also determined by trained personnel. Digesta from the pair of ceca were mixed and divided into two subsamples. One part was stored in plastic RNAse and DNAse free tubes, placed in liquid nitrogen, and afterward kept at −80 °C for analyses of gut microbiota. The other part was placed in bio-freeze kits (Alimetric Diagnostics, Espoo, Finland) for the determination of short-chain fatty acids.

### 2.4. Chemical Analyses

#### 2.4.1. Proximate Analysis

The OH and diet samples were finely ground and were all analyzed for CP, NDF, ADF, fat, and minerals. Dry matter was determined according to AOAC [27] method (925.09) by oven drying a 5.0 g sample at 105 °C overnight. Nitrogen content in samples was determined using the combustion method (990.03) [27] with an N analyzer (Model CNS-2000; LECO Corp., St. Joseph, MO, USA) and CP was calculated as N × 6.25. Ether extract in samples was determined after hexane extraction (Method 920.39) [27] in an Ankom extraction system (Macedon, NY, USA). Samples were analyzed for minerals after ashing at 600 °C for 12 h in a muffle furnace, using inductively coupled plasma mass spectrometry (ICP-AES; Vista, Varian, Palo Alto, CA, USA) according to the method of AOAC [28] (method 985.01). The ADF, NDF and lignin contents of samples were determined using the Ankom extraction system, which utilized the procedures of Goering and Van Soest [29]. Simple sugars were analyzed using the method Hall et al. [30]. Starch was determined using method 996.11 of AOAC [28]. Samples for non-structural carbohydrates were analyzed by Central Testing Laboratory, Winnipeg Manitoba using their standard operating procedures.

#### 2.4.2. Blood Biochemistry Analysis

Samples for blood biochemical analysis were centrifuged at 5000 rpm for 10 min and shipped on ice to Atlantic Veterinary College, University of Prince Edward Island Pathology Laboratory, where samples were analyzed using cobas^®^ 6000 analyzer series (Roche Diagnostics, IN, USA).

#### 2.4.3. Short-Chain Fatty Acids and Total Bacterial Density

Samples were immediately preserved using BioFreeze™ sampling kits (Alimetrics Diagnostics Ltd., Espoo, Finland) following the recommended protocol by the manufacturer. The analysis of dominant bacterial species as well as SCFA profiles was performed by Alimetrics Diagnostics Ltd. Samples were washed and centrifuged for collection of bacterial cells. The cell walls were then disrupted, and chromosomal DNA was extracted and purified for use in quantitative real-time PCR analyses. The SCFA profiles were analyzed by gas chromatography (Agilent Technologies, Santa Clara, CA, USA) using pivalic acid (Sigma-Aldrich, St. Louis, MO, USA) as an internal standard. The chromatography procedure which used a glass column packed with 80/120 Carbopack B-DA/4% Carbowax stationary phase, helium as a carrier gas, and a flame ionization detector has been described previously by Apajalahti et al. [31].

#### 2.4.4. Quantitative Real-Time PCR Analyses of Digesta DNA Samples

The qPCR system was based on the detection and quantification of a fluorescent reporter. This signal increases in direct proportion to the amount of PCR product in the reaction. By recording the amount of fluorescence emission at each cycle, it is possible to monitor the PCR reaction during exponential phase where the first significant increase in the amount of PCR product correlates to the initial amount of target template. For DNA extraction, PCR, sequencing, and sequence processing, specimens were placed into a MoBio PowerMag Soil DNA Isolation Bead Plate. DNA was extracted following MoBio’s instructions on a KingFisher robot. Bacterial 16S rRNA genes were PCR-amplified with dual-barcoded primers targeting the V4 region, as per the protocol of Kozich et al. [32]. Amplicons were sequenced with an Illumina MiSeq using the 300-bp paired-end kit (v.3). Sequences were denoised, taxonomically classified using Greengenes (v. 13_8) as the reference database, and clustered into 97%-similarity operational taxonomic units (OTUs) with the mothur software package (v. 1.39.5) [33], following the recommended procedure. The potential for contamination was addressed by co-sequencing DNA amplified from specimens and four each of template-free controls and extraction kit reagents processed the same way as the specimens. Two positive controls, consisting of cloned SUP05 DNA, were also included (number of copies = 2 × 106). Operational taxonomic units were considered putative contaminants (and were removed) if their mean abundance in controls reached or exceeded 25% of their mean abundance in specimens.

### 2.5. Bioinformatics and Statistical Analyses

Alpha diversity was estimated with the Shannon index on raw OTU abundance tables after filtering out contaminants. The significance of diversity differences was tested with an ANOVA. To determine beta diversity across samples, we excluded OTUs occurring with a count of less than three in at least 10% of the samples and then computed Bray-Curtis indices. We visualized beta diversity, emphasizing differences across samples, using Principal Coordinates Analysis (PCoA) ordination. Variation in community structure was assessed with permutational multivariate analyses of variance (PERMANOVA) with treatment group as the main fixed factor and using 4999 permutations for significance testing. High quality reads were classified using Greengenes v. 13_8 as the reference database. We aggregated OTUs into each taxonomic rank and plotted the relative abundance of the most abundant ones. All analyses were conducted in the R environment. DESeq2 package was used to identify differentially abundant taxa among variables. One-way Analysis of variance was carried out using the mixed procedure of SAS. The treatments Basal, BMD, COH, and EOH were the factors. The following parameters were the variables: feed intake, body weight, body weight gain, feed conversion ratio, organ weights, concentrations of SCFAs, and blood chemistry parameters. Normal probability plot of residuals was used to ascertain the normality of all data sets in the same statistical package. Datasets found to be non-normal were log base ten transformed. Lipase dataset was inverse transformed, while the total bilirubin dataset failed to achieve normality with transformation. Hence, a non-parametric Kruskal-Wallis test was used to test the effect of treatment on levels of blood total bilirubin acid. Following ANOVA, differences between significant means were tested using Tukey’s honest significant difference (HSD) test in the same statistical package. Analyzed data are presented as means, standard error of the means (SEM), and probability values. Values were considered statistically different at *p* ≤ 0.05.

## 3. Results

### 3.1. Effect of Coarse and Extruded OH on Growth Performance, Organ Weights, Cecal Short-Chain Fatty Acids and Blood Biochemical Parameters in Broiler Chickens

As illustrated in Table 1, the lignin content in the extruded OH increased by 28.7% compared to that of the coarse OH. On the other hand, the starch content in the extruded OH reduced by 38% compared to the coarse OH.

As presented in Table 3, there was no effect of OH on feed intake except during the starter phase when chickens fed COH had reduced (*p* = 0.0052) FI compared to the other treatments. Birds fed COH and EOH had reduced (*p* < 0.05) body weight compared to the basal and antibiotic diet, but there was no effect of treatment of FCR. As Table 4 illustrates, there was no effect of dietary treatments on the relative weights of bursa, spleen, and ceca. However, there was a significant increase (*p* = 0.0050) in relative gizzard weight among birds that were fed COH, compared to the other treatments. As presented in Table 5, there was no effect of dietary treatment on cecal short-chain fatty acids components, including acetic, propionic, butyric, lactic, and valeric acids. Table 6 shows that there was no effect of dietary treatment on blood biochemical parameters.

### 3.2. Effect of Coarse and Extruded OH on Gut Microbiota of Broiler Chickens

**Figure 1:**
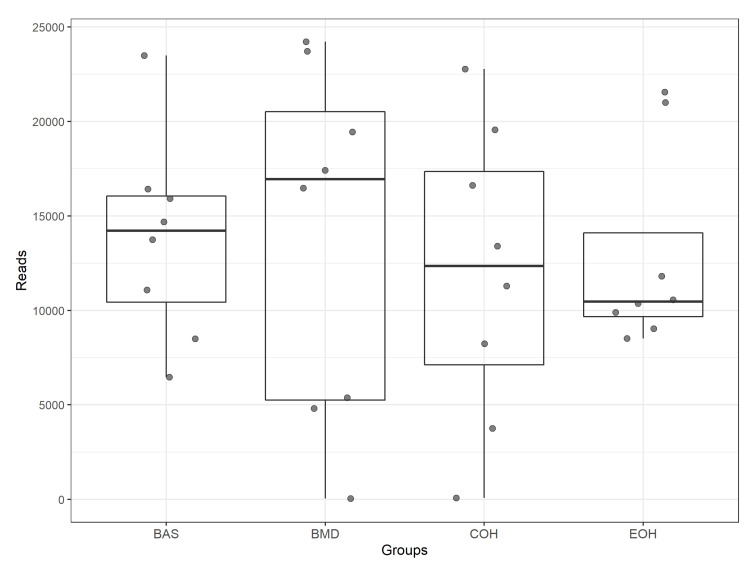
Box-and-whisker plot illustrating the total number of quality filtered per sample. These reads reflect the total number of high-quality sequences that align with 16Sv4, clustered into OTUs, and were assigned a taxonomic classification. BAS = basal diet; BMD = antibiotic diet; COH = diet supplemented with 3% coarse oat hulls; and EOH = diet supplemented with 3% extruded oat hulls.

**Figure 2:**
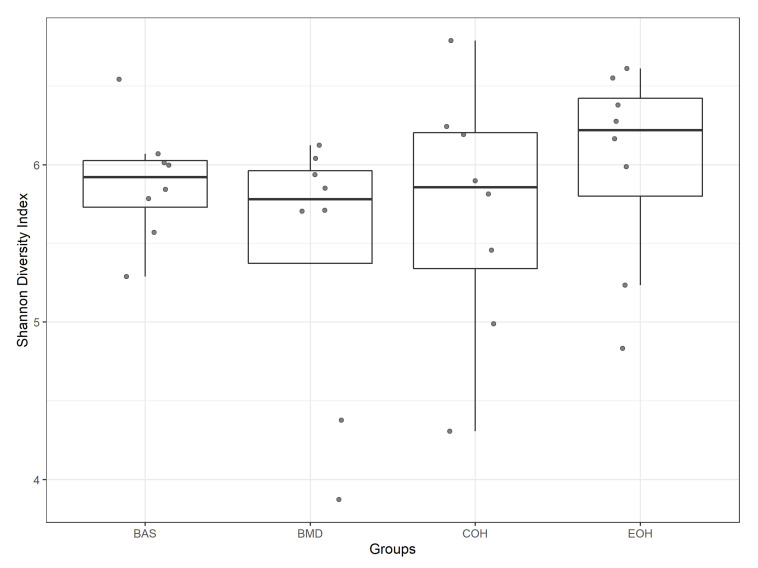
Alpha diversity (Shannon index) was computed and illustrated for each sample. BAS = basal diet; BMD = antibiotic diet; COH = diet supplemented with 3% coarse oat hulls; and EOH = diet supplemented with 3% extruded oat hulls.

**Figure 3:**
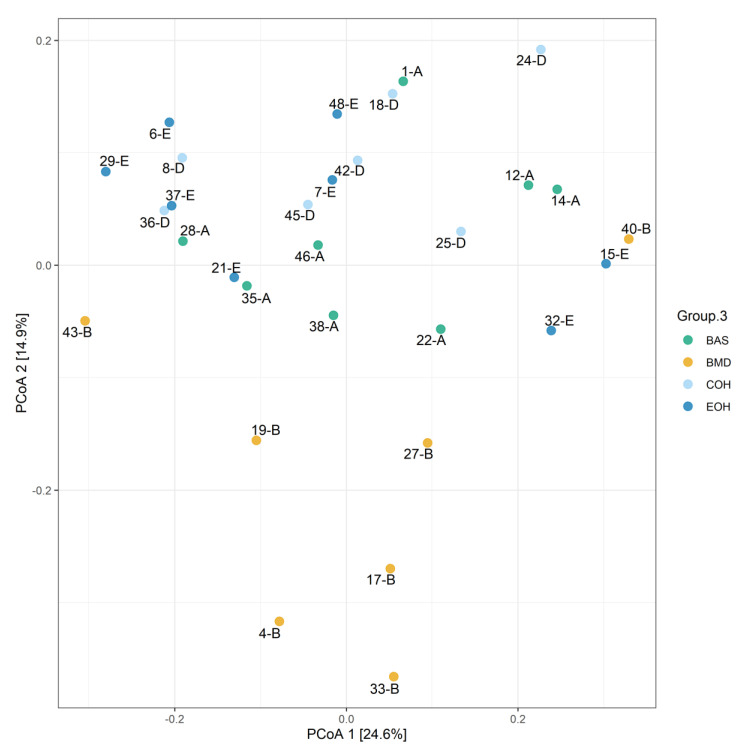
pCoA ordination performed by Bray-Curtis dissimilarities showing the microbiome composition for all specimens. BAS = basal diet; BMD = antibiotic diet; COH = diet supplemented with 3% coarse oat hulls; and EOH = diet supplemented with 3% extruded oat hulls.

**Figure 4:**
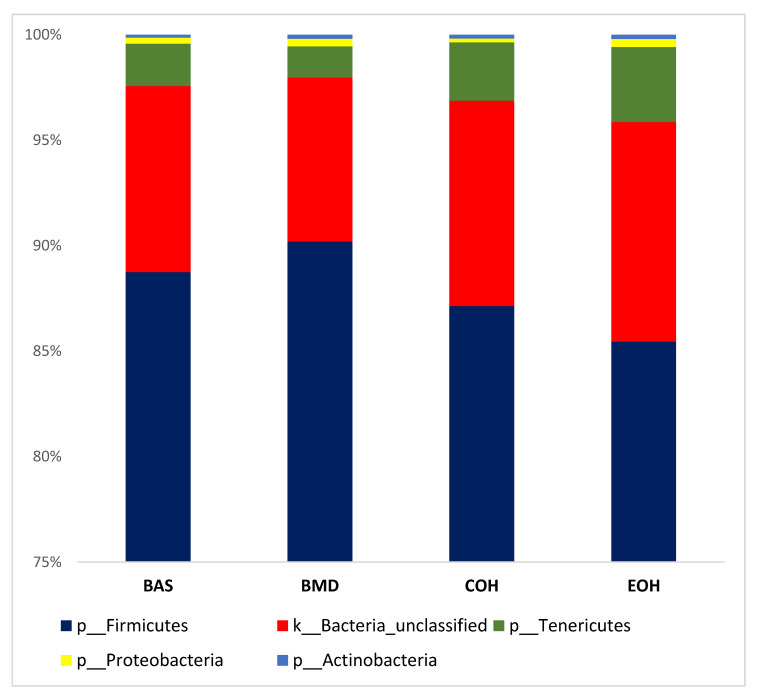
Illustration of the mean and standard error of the relative abundances of the most abundant Phyla. BAS = basal diet; BMD = antibiotic diet; COH = diet supplemented with 3% coarse oat hulls; and EOH = diet supplemented with 3% extruded oat hulls.

**Figure 5:**
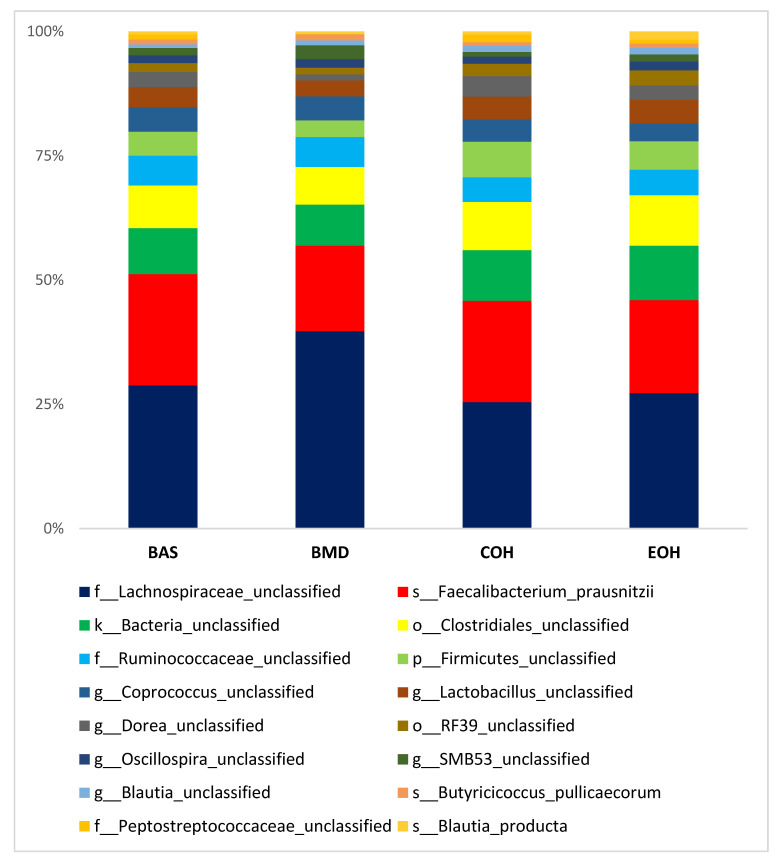
Illustration of the mean and standard error of the relative abundances of the most abundant species-level taxa within the four most abundant Phyla. The groupings along the y-axis represent the column of metadata. BAS = basal diet; BMD = antibiotic diet; COH = diet supplemented with 3% coarse oat hulls; and EOH = diet supplemented with 3% extruded oat hulls.

**Figure 6:**
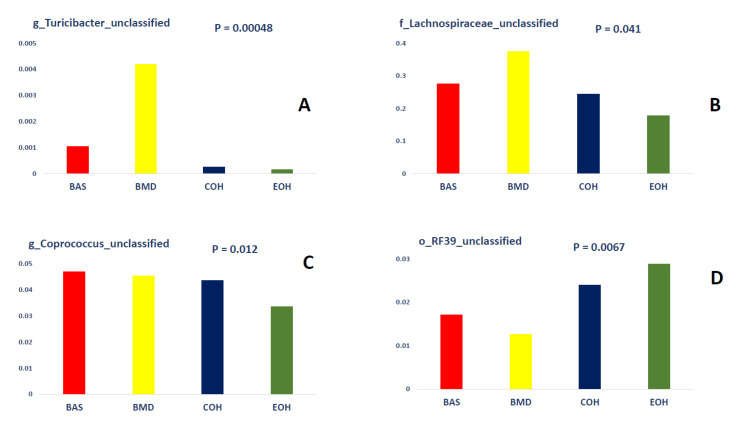
Illustration of the explanatory taxa for g_Turicibacter (**A**), f_Lachnospiracea (**B**), g_Coprococcus (**C**), and o_RF39_unclassified (**D**). BAS = basal diet; BMD = antibiotic diet; COH = diet supplemented with 3% coarse oat hulls; and EOH = diet supplemented with 3% extruded oat hulls.

**Figure 7:**
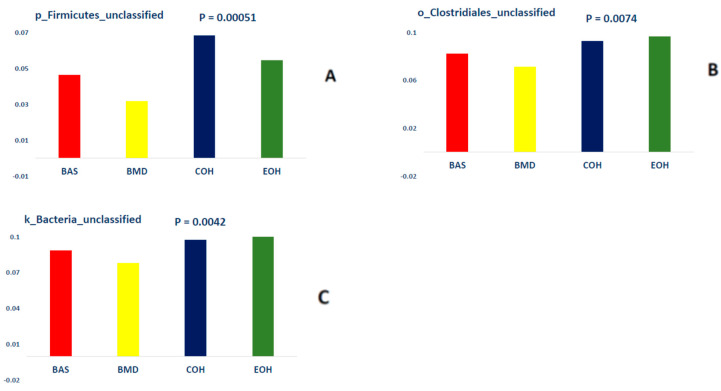
Illustration of the explanatory taxa for p_Firmicutes (**A**), o_Clostridilaes (**B**), and k_Bacteria_unclassified (**C**). BAS = basal diet; BMD = antibiotic diet; COH = diet supplemented with 3% coarse oat hulls; and EOH = diet supplemented with 3% extruded oat hulls.

A total of 53,470 OTUs were detected, with an average of 13,135 quality-filtered reads generated per sample. There was no effect of dietary treatment on read counts, as illustrated in Figure 1. There was no effect of dietary treatment on the Shannon diversity index in the number of species (OTUs at 97% similarity), which is a measure of richness and evenness in the samples (Figure 2). The pCoA ordination shows a distinctive clustering (Figure 3) of BMD, being lower than other treatment groups. Cecal microbial composition of chickens was mostly comprised of Firmicutes, Bacteria unclassified, and Tenericutes. The other less abundant phyla were Proteobacteria and Actinobacteria. Microbial communities at the phylum, class, and family levels were not influenced by dietary treatments (Figure 4). Differential abundance testing identified seven OTUs at the species level that were differentially abundant among treatments (Figure 5). The taxa affected by COH and EOH supplementation were illustrated in Figure 6 and Figure 7. All the differentially abundant OTUs, which are mainly from the Phyla Firmicutes and Tenericutes, are as follows: (1) g_Turicibacter_unclassified (Phylum Firmicutes) was significantly reduced (*p* = 0.00048) in the COH and EOH groups; (2) p_Firmicutes_unclassified (Phylum Firmicutes) was significantly reduced (*p* = 0.0051) in the BMD and EOH groups; (3) k_Bacteria_unclassified (Phylum Bacteria unclassified) was significantly increased (*p* = 0.0042) in the COH and EOH groups; (4) o_RF30_unclassified (Phylum Tenericutes) was significantly reduced (*p* = 0.0067) in the BMD, COH and EOH groups; (5) o_Clostridiales_unclassified (Phylum Firmicutes) was significantly reduced (*p* = 0.0074) in the BMD and EOH groups; (6) g_Coprococcus_unclassified (Phylum Firmicutes) was significantly increased by EOH treatment; and (7) f_Lachnospiraceae_unclassified (Phylum Firmicutes) was significantly increased (*p* = 0.041) in the COH and EOH groups.

## 4. Discussion

In Canada, about 3.3 million tons of oat is produced annually [34]. It is believed that OH accounts for 25% of oats [35]; therefore, about 0.8 million tons of OH is being generated annually in Canada. A large percentage of OH goes to waste [36], making OH a viable fiber source for poultry. Most of the chemical compositions of the coarse and extruded OH used in the current study compared favorably with that of the fine OH used in our previous study [8]. There were significant alterations in the contents of lignin and starch by extrusion process. A previous study has reported that physical processing altered the lignin content in oat hulls [36]. Oat hulls are considered a lignocellulose biomass, majorly consisting of lignin (16%), hemicellulose (16%) and cellulose (48%) on dry weight basis [36]. It has been noted that physical processing disrupts the bonds between the lignin, cellulose and hemicellulose in OH [36], and this could explain the reason for the increased lignin content in extruded OH used in the current study. Adewole et al [37] also reported that heat treatment may result in increased lignin content and reduced contents of simple sugars of feed ingredients as a result of Maillard reaction. This increased lignin content may be due to the formation of polymers called melanoidins (a Maillard reaction product) [38], which contribute to the analyzed lignin content [37]. 

The current study showed a reduction in body weight gain without a negative effect on feed conversion ratio, when 3% coarse or extruded OH were included to replace corn in broiler chickens’ diet. Previous research has shown that birds fed coarse diets performed better than those given fine particle-sized diets [39] and that coarse diet increased the efficiency of nutrient retention in broiler chickens [40]. In our previous study [8], the inclusion of 3% fine OH (isocaloric and isonitrogenous with the basal diet) did not have a negative effect on growth performance and carcass weight in broiler chickens. The reduction in body weight gain observed in the current study can be attributed to the fact the OH diets had reduced energy content, compared to the basal and BMD diets. Interesting to note is the fact that despite the energy dilution by the coarse and extruded OH in the current study, there was no adverse effect on feed conversion ratio, showing that farmers could save money due to OH addition in their nutrition program. In poultry production, feed cost represents a major portion of about 70% of the total cost. Dietary energy sources such as cereal grains occupy the greatest portion of the diets (70–75%) [41].

Consumption of coarse OH can be challenging for young chicks, and this could explain why feed intake was reduced in the COH group but not in the EOH group, during the starter phase. The current study observed higher gizzard weight in birds fed COH but not in those fed EOH, compared to the basal and BMD diets. The inclusion of large particle-sized fiber helps the gizzard to function optimally, reducing the particle size of ingested feed and mixing them with digestive enzymes [42,43,44]. The inclusion of at least 20–30% cereal particles larger than 1 mm or the inclusion of at least 3% fiber in the diet has been recommended by Svihus [45], to stimulate gizzard activity and to maximize broiler performance. Dietary inclusion of the coarse component of whole wheat increased gizzard weight and stimulated pancreatic enzyme activities in the small intestine of broiler chickens [46]. It has been reviewed by Amerah et al. [47] that feeding coarse particle-sized ingredients helps to reduce the energy cost of grinding in feed mills. When coarse particle-sized ingredients are fed to animals, an added benefit of energy savings in grinding in the feed mill is obtained [48]. Reece et al. [39] observed that 27% energy savings might be achieved when sieve size in hammer mills increases from 4.76 to 6.35 mm. The lack of effect of EOH on gizzard weight could be related to the reduced particle size and redistribution of the fiber components by extrusion process.

There was no effect of coarse or extruded OH on blood biochemical parameters in the current study. The influence of different forms of dietary fiber on blood lipid levels has been previously recognized [49,50]. Sarikhan et al. [51] investigated the effect of insoluble fiber on blood biochemical characteristics of broiler chickens and observed significant reduction in levels of triglycerides and cholesterol compared to the control, but no effect on total protein, albumin, amylase, lipase, and phosphorous. McNaughton [52] reported a significant reduction in plasma cholesterol when fiber ingredients, including alfalfa meal, oats, rice mill feed, and wood shavings, were fed to laying hens, and this was attributed to the hypocholesterolemic properties of the bioactive compounds such as saponins, present in those ingredients. This effect can also be related to the reduction in the concentration of bile acids, due to the binding effect of nonstarch polysaccharides [53] and the capacity of nonstarch polysaccharides to increase bile acid excretion [54]. Plausible factors that can be responsible for lack of effect of fiber on blood biochemical parameters in the current study include the low level of OH inclusion and shorter duration of feeding as some of the previous studies [35,36] had their trials lasting for 42 days or more. Blood biochemical parameters are useful tools for identifying metabolic maladies and subclinical ailments that can alter productivity, and the lack of effect in the current study implies that the health status of the birds was not affected by OH inclusion [55].

Dietary supplementation with COH and EOH did not significantly increase cecal SCFA production. However, there were numerically higher contents of acetic acid, butyric acid, and total SCFA in the ceca of birds fed COH. Similar results were obtained when broiler chickens’ diet was supplemented with 3% fine OH [8] and 3% sunflower hulls [56]. Most SCFA produced in the gut results from the fermentation of complex carbohydrates [56]. The lack of significant effect of OH inclusion in the current study and other previous studies could be as a result of low inclusion levels, which did not provide sufficient or sustained substrate for bacteria fermentation. In the study of Leung et al. [57], who fed 33% OH to broiler breeders, an increase in the concentration of acetic acid and total SCFA was observed compared to the control diet. The lack of treatment effect on SCFA production in the current study also suggested that not many SCFA producers were favored by OH supplementation either as coarse or extruded form.

Diet is an essential determinant of gut microbiota composition because it provides food substrates for gut microorganisms [58,59]. Both feed ingredients and their physical forms have been reported to modulate gut microbiota [60,61]. Similar to what was observed in the current study, previous studies have reported that Firmicutes and Bacteroides are the dominant phyla in the chicken cecal microbiota [62,63,64]. Our study showed a distinctive clustering of the BMD treatment in the lower axis of the pCoA ordination, compared to other treatment groups. This observation indicates a reduction in microbial diversity and relative abundance of intestinal bacteria in the BMD treatment group. One of the mechanisms of action of antibiotics is to modify intestinal microbial diversity and relative abundance [65]. In-feed antibiotics, including BMD, salinomycin, virginiamycin and tylosin have been reported to reduce the composition of chicken gut microbiota [66,67,68]. This reduction did not exclude the beneficial commensal bacteria [69,70,71]. This finding implies that the use of in-feed antibiotics is associated with a negative effect of reducing the microbiota population, including the beneficial ones.

Dietary treatments did not influence microbial communities at the phylum level in the current study. However, there were some differences at lower taxonomic levels. This trend has also been observed in previous studies [63,72]. An unclassified species within the genus Turicibacter was significantly reduced by dietary supplementation with COH and EOH groups (Figure 6). Turicibacter has been reported to be relevant to disease infections in mammalian studies [73,74]. The only species of Turicibacter that has been thoroughly described in the literature was Turicibacter Sanguinis, which was isolated from the blood culture of a febrile patient with acute appendicitis [73]. Turicibacter is correlated with Escherichia, Hafnia, and Shigella and is capable of altering normal host physiology and feeding behavior in chickens [75]. Other studies have found a negative correlation between Escherichia/Shigella and nutrient digestibility and weight gain in chickens [76]. In another broiler chicken study involving a Salmonella challenge, the abundance of Turicibacter was significantly increased by the Salmonella challenge [77]. It has been specified that Turicibacter sanguinins may cause subclinical infection or have some detrimental effects on the gastrointestinal tract [78]. In a study with rats, Turicibacter showed a tendency to reduce when a high fiber (barley) diet was fed, compared to those fed a high-fat diet [79]. Also, the abundance of the Turicibacter genus has been associated with a lack of physical exercise in studies with rodents [80,81]. An unclassified specie in the genus Lachnospiraceae was significantly increased in the cecal microbiota of birds fed COH and EOH diets (Figure 6). However, the unclassified species in the genus Coprococcus was significantly reduced in the cecal microbiota of only the birds fed EOH diet (Figure 6). This differential effect could possibly be associated with the modification of fiber fractions, which could be selectively fermentable by certain cecal bacteria, due to extrusion cooking of OH. Extrusion cooking may have also resulted in a high proportion of disruption in ligno-cellulose bonds. Lignocellulose degrading bacteria are limited in the cecum but are abundant and grow rapidly in the rumen of animals that feed on low quality roughages and agricultural and industrial wastes [82]. Extrusion process increased lignin content, potentially resulting from the contribution of melanoidins to the analyzed lignin content. Melanoidins have been recognized as beneficial to human health [83] because of their prebiotic properties [84]. It has been demonstrated that melanoidins are fermentable by resident gut bacteria in humans [85]. Lachnospiraceae are firmicute family that produce SCFA, particularly butyrate [86]. Other butyrate producers include feacelibacterium, Eubacterium, Coprococcus, Anaerostipes, and Lactobacilli. Lachnospiraceae has been found to dominate the fecal bacteria of healthy human adults [87]. They have been referred to as early colonizers of the chicken gastrointestinal tract. For example, in the study of Richards et al. [88], which determined the development of cecal microbiota in broiler breeds, Lachnospiraceae was the first Clostridia to colonize the ceca. Members of the Lachnospiraceae family can degrade complex polysaccharides to produce butyrate [89]. Similar to our findings, Munyaka et al. [72] reported that the family Lachnospiracea and other members of clostridium are some of the main bacterial components of the chicken cecum. A positive correlation has been found between Lachnospiraceae abundance and feed conversion ratio in broiler chickens [90]. Both Coprococcus and Lachnospiraceae were found to be positively correlated with appendicular muscle mass index in a human study [91]. The COH and EOH treatments significantly increased unclassified members of order Clostridiales. In previous studies, enrichment of the cecal digesta with bacteria belonging to the order Clostridiales has been reported when several antibiotics alternatives were fed to chickens [67,92,93]. Some studies have suggested that these microorganisms could be used as in-feed probiotics for poultry [90,94,95]. Moreover, members of the order Clostridiales are mostly non-pathogenic commensal bacteria, which have been reported to be associated with the prevention of inflammatory bowel disease and maintenance of mucosal homeostasis [96]. Members of the order Clostridiales have also been associated with butyrate production in chickens [97].

## 5. Conclusions

Supplementation of broiler chickens’ diets with 3% COH or EOH reduced BWG without affecting FCR and modified few cecal microbiota species with no effect on SCFA concentration and blood biochemical parameters. Dietary supplementation with COH but not EOH significantly increased the relative gizzard empty weight of broiler chickens. Though supplementation of broiler chickens’ diets with COH or EOH may be favorable for the establishment of few beneficial cecal microbiota in the gut, EOH may differentially modulate few bacteria species, due to potential modification of the fiber fractions and possible disruption of lignocellulose bonds. However, more research is needed in this area to elucidate the effect of COH and EOH on microbiota in different sections of the chicken gastrointestinal tract 

## Figures and Tables

**Table 1:** Analyzed composition of the oat hulls used in the experiment (% as fed basis).

	Coarse Oat Hulls	Extruded Oat Hulls
Dry matter	90.3	92.5
Crude protein	2.52	2.92
Fat	0.50	0.55
Acid detergent fiber	42.3	40.3
Neutral detergent fiber	76.2	73.9
Lignin	6.62	9.29
Ash	5.45	4.99
Simple sugars	1.10	0.63
Starch	4.37	2.70
Non-structural carbohydrates	7.68	6.72
Calcium	0.07	0.07
Potassium	0.27	0.28
Magnesium	0.07	0.07
Phosphorus	0.06	0.07

**Table 2:** Ingredient and nutrient compositions of diets in the experiment ^1.^

Item	Production Period (Days of Age)											
	1–14				14–24				24–36			
	Basal	BMD	COH	EOH	Basal	BMD	COH	EOH	Basal	BMD	COH	EOH
Corn	41.3	41.2	38.0	38.0	44.3	44.2	40.9	40.9	48.5	48.4	45.1	45.1
Soybean Meal	40.2	40.2	40.5	40.5	36.5	36.5	36.9	36.9	31.5	31.5	31.9	31.9
Wheat	10.0	10.0	10.0	10.0	10.0	10.0	10.0	10.0	10.0	10.0	10.0	10.0
Oat Hulls	-	-	3.00	3.00	-	-	3.00	3.00	-	-	3.00	3.00
Animal/Vegetable Fat	3.44	3.47	3.40	3.40	4.59	4.63	4.59	4.59	5.67	5.70	5.67	5.67
Limestone	1.80	1.80	1.79	1.79	1.65	1.65	1.64	1.64	1.52	1.52	1.51	1.51
Dicalcium Phosphate	1.23	1.23	1.24	1.24	1.06	1.06	1.07	1.07	0.93	0.93	0.94	0.94
DL-Methionine Premix ^Z^	0.61	0.61	0.62	0.62	0.53	0.53	0.54	0.54	0.49	0.49	0.50	0.50
Lysine HCl	0.03	0.03	0.02	0.02	-	-	-	-	0.01	0.009	0.003	0.003
Iodized Salt	0.40	0.40	0.40	0.40	0.37	0.37	0.37	0.37	0.38	0.38	0.38	0.38
Pellet Binding Agent ^Y^	0.50	0.50	0.50	0.50	0.50	0.50	0.50	0.50	0.50	0.50	0.50	0.50
BMD 110 G ^X^	-	0.05	-	-	-	0.05	-	-	-	0.05	-	-
Vitamin/Mineral Premix ^W, V^	0.50	0.50	0.50	0.50	0.50	0.50	0.50	0.50	0.50	0.50	0.50	0.50
Calculated Composition												
Crude Protein (%)	23.0	23.0	23.0	23.0	21.5	21.5	21.5	21.5	19.5	19.5	19.5	19.5
Metabolizable Energy (MJ·kg^−1^)	12.6	12.6	12.2	12.2	13.0	13.0	12.6	12.6	13.4	13.4	13.0	13.0
Calcium (%)	0.96	0.96	0.96	0.96	0.87	0.87	0.87	0.87	0.78	0.78	0.78	0.78
Available Phosphorus (%)	0.48	0.48	0.48	0.48	0.44	0.44	0.44	0.44	0.39	0.39	0.39	0.39
Digestible Lysine	1.28	1.28	1.28	1.28	1.16	1.16	1.17	1.17	1.02	1.02	1.03	1.03
Digestible Methionine + Cystine (%)	0.95	0.95	0.95	0.95	0.87	0.87	0.87	0.87	0.80	0.80	0.80	0.80
Sodium (%)	0.19	0.19	0.19	0.19	0.18	0.18	0.18	0.18	0.18	0.18	0.18	0.18
Analysed composition ^u^												
Crude Protein (%)	24.6	23.9	24.9	24.0	21.8	22.1	22.6	21.3	20.4	20.2	20.9	20.4
ADF (%)	3.74	4.82	5.85	5.08	3.15	3.70	4.41	4.83	3.87	3.84	5.02	5.84
NDF (%)	7.26	7.33	9.16	7.64	6.81	6.69	8.59	8.86	7.44	7.27	8.17	9.79
Sodium (%)	0.20	0.19	0.21	0.20	0.19	0.18	0.17	0.17	0.18	0.19	0.19	0.18
Crude Fat (%)	5.55	5.35	5.50	5.44	6.90	6.93	7.02	6.85	7.61	7.91	7.96	7.91

^1^ Basal, basal diet, BMD, antibiotic diet, COH, diet containing 3% coarse oat hulls, EOH, diet containing 3% extruded oat hulls. ^z^ Supplied/kg premix: DL-Methionine, 0.5 kg; wheat middlings, 0.5 kg. ^Y^ Pel-stik: Uniscope, Inc., Johnstown, CO, USA. ^X^ Bacitracin methylene disalicylate (providing 55 mg/kg mixed feed); Alpharma, Inc., Fort Lee, NJ, USA. ^w^ Starter vitamin-mineral premix contained the following per kg of diet: 9750 IU vitamin A; 2000 IU vitamin D3; 25 IU vitamin E; 2.97 mg vitamin K; 7.6 mg riboflavin; 13.5 mg Dl Ca-pantothenate; 0.012 mg vitamin B12; 29.7 mg niacin; 1.0 mg folic acid, 801 mg choline;0. 3 mg biotin; 4.9 mg pyridoxine; 2.9 mg thiamine; 70.2 mg manganese; 80.0 mg zinc; 25 mg copper; 0.15 mg selenium; 50 mg ethoxyquin; 1543mg wheat middlings; 500 mg ground limestone. ^v^ Grower and Finisher vitamin-mineral premix contained the following per kg of diet: 9750 IU vitamin A; 2000 IU vitamin D3; 25 IU vitamin E; 2.97 mg vitamin K; 7.6 mg riboflavin; 13.5 mg Dl Ca-pantothenate; 0.012 mg vitamin B12; 29.7 mg niacin; 1.0 mg folic acid, 801 mg choline;0. 3 mg biotin; 4.9 mg pyridoxine; 2.9 mg thiamine; 70.2 mg manganese; 80.0 mg zinc; 25 mg copper; 0.15 mg selenium; 50 mg ethoxyquin; 1543mg wheat middlings; 500 mg ground limestone. ^u^ As fed basis.

**Table 3:** Effect of dietary supplementation with coarse or extruded oat hulls on growth performance of broiler chickens.

	Treatment ^1^				SEM	*p* Value
	Basal	BMD	COH	EOH		
Feed intake, g/bird						
D 1–14	542 ^a^	548 ^a^	489 ^b^	515 ^ab^	11.22	0.0052
D 14–28	1890	1857	1818	1821	32.37	0.3644
D 28–36	1165	1153	1206	1185	21.61	0.3439
D 1–36	3585	3587	3397	3522	77.07	0.2819
Body weight gain, g/bird						
D 1–14	447 ^a^	455 ^a^	408 ^b^	413 ^b^	6.880	<0.0001
D 14–28	1168 ^ab^	1177 ^a^	1106 ^bc^	1104 ^c^	16.62	0.0038
D 28–36	817	828	794	815	15.73	0.5042
D 1–36	2449 ^ab^	2460 ^a^	2308 ^b^	2332 ^b^	28.06	0.0008
Feed conversion ratio						
D 1–14	1.21	1.21	1.19	1.25	0.022	0.2207
D 14–28	1.62	1.58	1.65	1.65	0.024	0.1198
D 28–36	1.44	1.40	1.53	1.45	0.038	0.0956
D 1–36	1.50	1.46	1.47	1.51	0.018	0.1881

^1^ Basal = basal diet; BMD = antibiotic diet; COH = diet supplemented with 3% coarse oat hulls; and EOH = diet supplemented with 3% extruded oat hulls. In a row, means assigned different lowercase letters are significantly different, *p* < 0.05 (Tukey’s procedure). SEM = standard error of the mean.

**Table 4:** Effect of dietary supplementation with coarse or extruded oat hulls on organ weights of broiler chickens.

Item	Treatment ^1^				SEM	*p* Value
	Basal	BMD	COH	EOH		
Slaughter weight, kg	2.80	2.82	2.73	2.90	0.096	0.6677
Bursa, g/kg body weight	1.82	1.69	1.74	1.92	0.182	0.8283
Spleen, g/kg body weight	0.95	0.85	0.88	0.78	0.069	0.4405
Gizzard, g/kg body weight ^2^	11.7 ^b^	11.7 ^b^	14.4 ^a^	11.7 ^b^	0.601	0.0050
Ceca, g/kg body weight ^3^	3.62	4.20	3.59	3.98	0.251	0.2660

^1^ Basal = basal diet; BMD = antibiotic diet; COH = diet supplemented with 3% coarse oat hulls; and EOH = diet supplemented with 3% extruded oat hulls. In a row, means assigned different lowercase letters are significantly different, *p* < 0.05 (Tukey’s procedure). SEM = standard error of the mean. ^2^ Empty weight of gizzard ^3^ Empty weight of ceca.

**Table 5:** Effect of dietary supplementation with coarse or extruded oat hulls on cecal short chain fatty acids (SCFA) composition (mmol/kg) and total Eubacteria (16S rDNA copies/gram of sample) in broiler chickens.

Item	Treatment ^1^				SEM	*p* Value
	Basal	BMD	COH	EOH		
Acetic acid	51.5	51.2	55.6	51.1	4.330	0.8578
Propionic acid	4.29	5.26	4.15	4.36	0.459	0.3275
Butyric acid	12.2	12.3	13.5	10.4	1.328	0.4380
Valeric acid	0.94	1.06	1.03	0.97	0.081	0.7299
Lactic acid	3.00	1.58	2.33	2.38	0.615	0.2166
Total SCFA	74.2	71.3	78.6	71.0	5.672	0.7648
BCFA ^2^	2.23	1.38	2.01	1.81	0.227	0.4822
VFA ^3^	71.2	69.8	76.3	68.6	5.745	0.7925
Total Eubacteria	12.0	11.9	11.8	11.9	0.109	0.8495

^1^ Basal = basal diet; BMD = antibiotic diet; COH = diet supplemented with 3% coarse oat hulls; and EOH = diet supplemented with 3% extruded oat hulls. SEM = standard error of the mean. ^2^ Branched chain fatty acids. ^3^ Volatile fatty acids.

**Table 6:** Effect of dietary supplementation with coarse or extruded oat hulls on blood biochemical characteristics of broiler chickens.

	Treatment ^1^				SEM	*p* Value
	Basal	BMD	COH	EOH		
**Electrolytes and minerals, mmol·L^−1^**						
Sodium	152	154	159	164	9.484	0.8073
Potassium	5.76	5.09	5.56	5.65	0.356	0.5622
Chloride	111	112	116	120	7.982	0.8451
Calcium	2.68	2.76	2.89	2.92	0.108	0.3696
Phosphorus	1.77	1.59	1.68	1.70	0.143	0.8640
Magnesium	0.83	0.82	0.80	0.75	0.053	0.7413
**Metabolites, mmol·L^−1^**						
Urea	0.34	0.26	0.30	0.28	0.031	0.3451
Creatinine	2.00	2.75	2.00	2.25	1.096	0.9570
Glucose	14.7	15.1	15.9	15.9	0.835	0.6450
Cholesterol	2.94	3.41	3.13	3.01	0.236	0.6009
Iron	17.9	23.0	20.3	17.3	2.584	0.3977
Bile acids	17.0	14.0	17.9	17.4	2.481	0.6887
Uric acid	401	447	407	350	58.62	0.9169
Total Bilirubin	0.63	0.38	0.38	0.38	0.183	0.7079
**Enzymes, U·L^−1^**						
Amylase	410	483	516	417	67.72	0.4481
Alkaline phosphatase	3243	2722	2820	3316	599.9	0.6884
Creatine kinase	19,820	19784	18888	27667	4524	0.3751
Aspartate Aminotransferase	324	308	327	378	48.29	0.6416
Gamma-Glutamyl Transferase	10.0	11.9	13.5	10.9	1.329	0.3016
Lipase	22.0	17.8	19.1	17.0	3.143	0.6908
**Proteins, g·L^−1^**						
Total protein	28.0	28.4	29.4	28.3	2.215	0.9733
Albumin	12.0	12.1	12.5	11.8	0.917	0.9499
Globulin	16.0	16.3	16.9	16.5	1.344	0.9718

^1^ Basal = basal diet; BMD = antibiotic diet; COH = diet supplemented with 3% coarse oat hulls; and EOH = diet supplemented with 3% extruded oat hulls. SEM = standard error of the mean.
